# A239 MOTIVATIONS BEHIND CAM USE IN PATIENTS WITH CROHN'S DISEASE AND ULCERATIVE COLITIS

**DOI:** 10.1093/jcag/gwad061.239

**Published:** 2024-02-14

**Authors:** N K Klemm, R Trasolini, B Bressler, G Rosenfeld, Y Leung

**Affiliations:** Gastroenterology, The University of British Columbia, Vancouver, BC, Canada; Gastroenterology, The University of British Columbia, Vancouver, BC, Canada; Gastroenterology, The University of British Columbia, Vancouver, BC, Canada; Gastroenterology, The University of British Columbia, Vancouver, BC, Canada; Gastroenterology, The University of British Columbia, Vancouver, BC, Canada

## Abstract

**Background:**

Complementary and alternative medicine (CAM) use is common in IBD patients and impacts compliance with conventional treatment. Gastroenterologists should understand the motivational factors of CAM use—factors that *push* patients away from standard therapy or *pull* towards CAM. Our study describes the motivations behind CAM use for IBD and evaluates differences between CD and UC patients.

**Aims:**

To compare the motivations behind CAM use between CD and UC patients.

**Methods:**

Retrospective cohort survey of patients over 18 years old with IBD, evaluated by gastroenterologists at a tertiary care referral centre from January 1 to December 31, 2019. Only patients that reported CAM use were included. Chi-square and independent t-tests were performed and p-value ampersand:003C0.05 was significant.

**Results:**

Of the 230 completed surveys, 193 reported CAM use (CD:57.5% & UC:42.5%). Demographics, disease duration and hospitalizations were similar, but CD patients had lower SIBDQ scores (CD:48.1 & UC:53.5 pampersand:003C0.001).

Both groups were largely influenced by their social network to use CAM (CD:33% & UC:31.3%) and did not feel well informed of CAM (87.4%). CD and UC patients had similar push and pull factors. Push factors included lack of improvement (39%) and side effects (20%) with conventional treatment. Pull factors included the desire for a holistic approach (21%) and to improve mood (35%). Most patients hoped fatigue 62.7%, diarrhea 61.7% and abdominal pain 58.0% would improve with CAM.

**Conclusions:**

Despite differences in QoL, push and pull motivations for CAM use do not differ between CD and UC patients. Most users do not feel well informed of CAM and ongoing dialogue is important for patient-centred care.

Table 1: Characteristics of CAM use

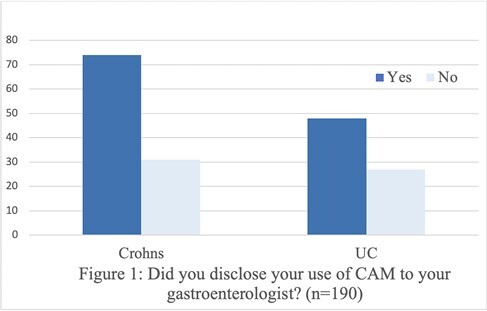

**Funding Agencies:**

None

**Table:** 

	CD	UC	p-value
How did you decide to use CAM? n=187 (%)			0.621
Advice from colleague, friend, family	36 (33.6)	25 (31.3)	
Physician	27 (25.2)	15 (18.8)	
Online	14 (13.1)	12 (15.0)	
How well informed do you feel about CAM for IBD? n=193 (%)			0.0674
Very informed	14 (7.4)	10 (5.3)	
Somewhat informed	58 (30.5)	47 (24.7)	
Not informed	30 (20.0)	23 (12.1)	
What are the reasons for using CAM? n=193 (%)			
Conventional medicine did not make me feel better	46 (41.4)	29 (35.4)	0.392
Conventional medicine will not cure my disease	24 (21.6)	14 (17.1)	0.432
Side effects of conventional treatment	22 (19.8)	17 (20.7)	0.876
Final hope	15 (13.5)	11 (13.4)	0.984
Fear of surgery	20 (18.0)	9 (11.0)	0.176
Want a natural approach	17 (15.3)	22 (26.8)	0.049
Want a holistic approach	22 (19.8)	18 (22.0)	0.718
Improve mood	41 (36.9)	26 (31.7)	0.451
What symptoms were you hoping would improve with CAM? n=193 (%)			
Abdominal Pain	69 (62.2)	43 (52.4)	0.176
Diarrhea	64 (57.7)	55 (67.1)	0.184
Fatigue	72 (64.9)	49 (59.8)	0.468
Appetite	28 (25.2)	11 (13.4)	0.043
Weight Loss	24 (21.6)	14 (17.1)	0.432

